# Sotrovimab: A Review of Its Efficacy against SARS-CoV-2 Variants

**DOI:** 10.3390/v16020217

**Published:** 2024-01-31

**Authors:** Daniele Focosi, Arturo Casadevall, Massimo Franchini, Fabrizio Maggi

**Affiliations:** 1 North-Western Tuscany Blood Bank, Pisa University Hospital, via Paradisa 2, 56124 Pisa, Italy; 2 Department of Molecular Microbiology and Immunology, Johns Hopkins Bloomberg School of Public Health, Baltimore, MD 21205, USA; acasade1@jhu.edu; 3 Department of Transfusion Medicine and Hematology, Carlo Poma Hospital, 46100 Mantua, Italy; massimo.franchini@asst-mantova.it; 4 National Institute for Infectious Diseases “Lazzaro Spallanzani” IRCCS, 00149 Rome, Italy; fabrizio.maggi@inmi.it

**Keywords:** sotrovimab, VIR-7831, S-309, SARS-CoV-2, efficacy, safety

## Abstract

Among the anti-Spike monoclonal antibodies (mAbs), the S-309 derivative sotrovimab was the most successful in having the longest temporal window of clinical use, showing a high degree of resiliency to SARS-CoV-2 evolution interrupted only by the appearance of the BA.2.86* variant of interest (VOI). This success undoubtedly reflects rational selection to target a highly conserved epitope in coronavirus Spike proteins. We review here the efficacy of sotrovimab against different SARS-CoV-2 variants in outpatients and inpatients, discussing both randomized controlled trials and real-world evidence. Although it could not be anticipated at the time of its development and introduction, sotrovimab’s use in immunocompromised individuals who harbor large populations of variant viruses created the conditions for its eventual demise, as antibody selection and viral evolution led to its eventual withdrawal due to inefficacy against later variant lineages. Despite this, based on observational and real-world data, some authorities have continued to promote the use of sotrovimab, but the lack of binding to newer variants strongly argues for the futility of continued use. The story of sotrovimab highlights the power of modern biomedical science to generate novel therapeutics while also providing a cautionary tale for the need to devise strategies to minimize the emergence of resistance to antibody-based therapeutics.

## 1. Introduction

Sotrovimab, also known as VIR-7831 or GSK-4182136 (Xevudy^®^, manufactured by GSK) [1,2] is a monoclonal antibody (mAb) derived from S-309 (an mAb isolated from a SARS-CoV convalescent) which targets a highly conserved epitope of the receptor-binding domain (RBD) within the Spike protein of SARS-CoV-2. In this regard, it is noteworthy that its origin was an antibody made for a coronavirus other than SARS-CoV-2 and that sotrovimab was chosen for clinical development based on powerful in vitro antiviral activity and because it targeted a relatively invariant epitope shared by two coronaviruses. Sotrovimab was classified as either an RBD core cluster I [3] or a class 3 mAb [4], binding to both the “up” and “down” conformation of the RBD and interacting with a unique proteoglycan epitope at residue N343 [5]. Sotrovimab works by inducing both neutralization of virus infection and antibody-dependent cell cytotoxicity (ADCC) [6]. Sotrovimab serum half-life was improved by inserting the Met428Leu/Asn434Ser (LS) mutation (Xtend^TM^) in the Fc region. Unlike with other half-life extended anti-Spike mAbs, this mutation does not impact the ADCC functions of sotrovimab, which are important for its activity against SARS-CoV-2 [7,8,9]. This mutation was previously used in the mAb ravulizumab (approved for paroxysmal nocturnal hemoglobinuria). Hence, sotrovimab was rationally chosen in the early days of the pandemic with the view that it may be more resilient to viral evolution by targeting a conserved domain and then enhance by molecular biology techniques for a longer serum half-life while preserving critical antiviral Fc functions. Having been authorized by the FDA for treatment of high-risk outpatients since 26 May 2021, sotrovimab has, to date, shown the highest levels of resilience in in vitro activity against SARS-CoV-2 sublineages except for the recently emerged BA.2.86* variant of interest (VOI) (“Pirola clan”) [10,11]. In this review we will discuss the results achieved by sotrovimab in clinical trials and post-marketing experiences and draw lessons from this experience that could help in the future design of mAb-based therapeutics. Given that the overall safety of anti-Spike mAbs has been excellent, we will focus on efficacy only.

## 2. Methods

A search of the literature in the PubMed (through Medline), EMBASE, Cochrane central, medRxiv, and bioRxiv databases of articles published and posted between 1 December 2019 and 29 December 2023 was carried out using English language as a criterion for selection. A search of the literature through MEDLINE and PubMed electronic databases was performed for articles published during the same timespan using the following Medical Subject Heading (MeSH) and query: (“COVID-19” OR “SARS-CoV-2”) AND “monoclonal antibody” AND “Spike” AND (“S-309” OR “sotrovimab” OR “VIR-7831”). We also screened the reference lists of the most relevant review articles for additional studies not captured in our initial search of the literature. 

## 3. Results

### 3.1. Outpatient RCT Efficacy

The efficacy of sotrovimab was established by the COMET-ICE double-blind randomized clinical trial (RCT) (NCT04545060) which evaluated it in unvaccinated patients at risk for progression, mostly from the USA and with symptoms for less than 5 days, between August 2020 and March 2021 (with infecting sublineages being a cocktail of Alpha, Epsilon, Gamma, and Zeta [12]). In this RCT, 500 mg of i.v. sotrovimab reduced hospitalization from 7% to 1% in an interim analysis on 583 patients [13], and the final results on 1057 patients confirmed a reduction from 6% to 1% in hospitalization lasting longer than 24 h or death at day 29 [14]. It is noteworthy that other anti-Spike mAbs and COVID-19 convalescent plasma (CCP) have also shown efficacy when administered early in the course of disease which, together with the sotrovimab results, makes a compelling case that antibody-based therapeutics are very effective in reducing the progression of COVID-19 [15]. Consistent with this notion, the MANTICO RCT in Italy (NCT05205759) found that among adult outpatients with mild-to-moderate SARS-CoV-2 infection due to Omicron BA.1 and BA.1.1, early treatment with sotrovimab reduced the time to recovery compared with casirivimab/imdevimab and bamlanivimab/etesevimab (mAbs that were both deauthorized by the FDA at that time because of inefficacy and which may thus represent an inadequate control arm) [16]. More recently, a small-sized RCT in Thailand comparing sotrovimab to the combination of CCP and favipiravir showed comparable efficacy for both regimens when used in outpatients with COVID-19 [17].

Sotrovimab is also being investigated in a Phase II trial (NCT05210101) on pre-exposure prophylaxis in 93 seronegative immunocompromised individuals [18].

### 3.2. Inpatient RCT Efficacy

In a multicenter TICO double-blind RCT (NCT04501978) involving 546 unvaccinated patients (mostly from hospitals in the USA) with more than 12 days of symptoms carried out between December 2020 and March 2021 (hence at the time of the B.1.2 and Epsilon VOC), sotrovimab did not reduce pulmonary complications on day 5 nor lead to better clinical recovery on day 90 than the placebo [19]. It is noteworthy that sotrovimab, like other anti-Spike mAbs, has not been shown to reduce mortality in inpatients. This is distinct from the results of CCP, which reduces mortality in hospitalized patients when used early in hospitalization with units that have high neutralizing antibody titers [20], including mechanically ventilated patients [21]. This may reflect some fundamental differences between the efficacy of monoclonal and polyclonal preparations in more advanced disease.

### 3.3. Viral Evolution and Baseline SARS-CoV-2 Susceptibility to Sotrovimab

**Figure 1:**
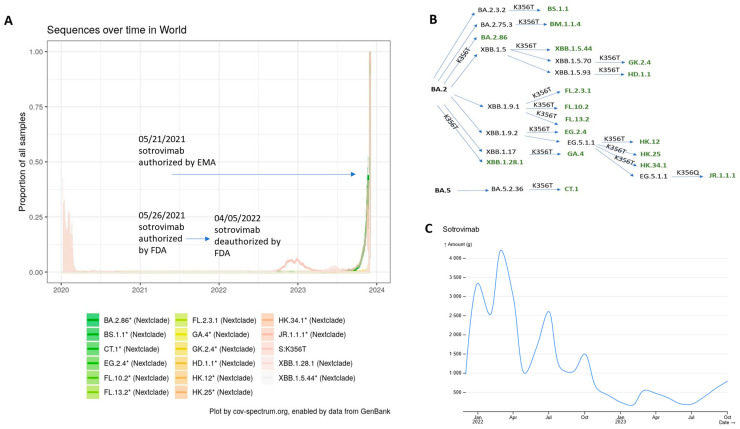
(**A**) Prevalence of the spike K356T mutation in worldwide SARS-CoV-2 sequences before and after the advent of BA.2.86* and its relationship with sotrovimab regulatory status. The early 2020 peak represents a large confidence interval because of the low number of viral sequences deposited. (**B**) All sublineages designated by PANGOLIN which acquired K356T (detailed on the top right part) are plotted, with JN.1 representing the current peak. The figure was generated using CoV-Spectrum [72]. (**C**) Absolute number of sotrovimab prescriptions in England (available at https://hospitalprescriptions.genomium.org/?searchTerm=sotrovimab&selectedMedication=%7B%22isid%22%3A%221162689008%22%2C%22nm%22%3A%22Sotrovimab%22%7D&mode=Ingredients&breakdownBy=none&plotType=smoothline on 15 January 2024) is reported in the lower right part.

Sotrovimab remained strongly active in vitro until BA.2, but its activity, as assessed by IC_50_ in in vitro viral neutralization assays on replication-competent cell lines, declined since the emergence of BA.4/5 (Table 1). Most importantly, binding to and viral neutralization efficacy were totally abolished by the emergence of the 2023 FLip’s lineages [22] and in BA.2.86* (“Pirola clan”) [23]. In the latter, the Spike mutation K356T creates a motif for glycosylation of N354 which abolishes sotrovimab binding to Spike. It is notable that this mutation, virtually absent before the marketing of sotrovimab, has become apparent in multiple sublineages since then but never reached significant prevalence before BA.2.86* (Figure 1). Table 2 summarizes the key SARS-CoV-2 Spike mutations that confer in vitro resistance to sotrovimab.

### 3.4. Sotrovimab Treatment-Emergent Immune Escape

A total of 12 case series have reported an incidence of sotrovimab treatment-emergent resistance; this resistance has ranged from 16 to 100%, with an overall incidence of 33% (146 out of 439 reported cases [24,25,26,27,28,29,30,31,32,33,34]). In comparison, in series with dual anti-Spike mAbs, treatment-emergent resistance ranged from 0% to 50% [35] (Table 3). For sotrovimab, the generated footprint is so unique (S:P337R/L and S:E340A/K/V) that baseline sequencing is not even needed to confirm emergence after treatment. In fact, the prevalence of any of these mutations has, to date, remained exceedingly rare in the GISAID database (assessed using CoV-Spectrum.org) during the pandemic (<0.1% global prevalence out of >15.4 million SARS-CoV-2 sequences on 15 January 2024).

While only 1 of the 35 patients in the COMET-ICE trial who had treatment-emergent resistance mutations experienced progression to hospitalization lasting longer than 24 h or death through day 29 [12], it should be noted that the COMET-ICE RCT did not recruit severely immunocompromised patients [14]. Severely immunocompromised patients have been the primary focus of sotrovimab treatment in real life and have a much higher risk of treatment-emergent resistance.

### 3.5. Real-World Evidence

Given that placebo-controlled RCTs are no longer considered ethical by most investigators, the only current sources of clinical efficacy data are standard-of-care-controlled RCTs or observational studies. The latter are mostly retrospective in nature and often lack propensity-score matched controls. During the Delta wave, Aggarwal et al. in Colorado matched 522 patients receiving sotrovimab to 1563 not receiving mAbs and demonstrated a 63% decrease in the odds of all-cause hospitalization (raw rate of 2.1% vs. 5.7%) and an 89% decrease in the odds of all-cause 28-day mortality (raw rate of 0% vs. 1.0%) [36]. These data were confirmed by Ong et al. in Singapore, who found that sotrovimab protected against in-hospital deterioration (hazard ratio, 0.41) [37]. On the other hand, Aggarwal et al. in Colorado reported that sotrovimab treatment was not associated with reduced odds of 28-day hospitalization (2.5% vs. 3.2%) or mortality (0.1% vs. 0.2%) during the BA.1 and BA.1.1 waves [38], for which sotrovimab had IC50 above 150 ng/mL (Table 1).

In a study conducted during the time period corresponding to the Delta and BA.1 waves in California, Cheng et al. found that a sotrovimab cohort had a 55% lower risk of 30-day hospitalization or mortality (RR 0.45) and an 85% lower risk of 30-day mortality than a no-mAb cohort (n = 1,514,868) (RR 0.15) [39]. Similar data were reported from Wales, where Evans et al. reported that in higher-risk adult patients in the community with COVID-19, those who received treatment with molnupiravir (n = 359), nirmatrelvir-ritonavir (n = 602), or sotrovimab (n = 1079) had lower risk of hospitalization or death than those not receiving treatment (n = 4973); there was no difference reported between the BA.1 and BA.2 waves [40].

In routine care of non-hospitalized high-risk adult patients with COVID-19 in England, no substantial difference in the risk of severe COVID-19 outcomes was observed between those who received nirmatrelvir/ritonavir (n = 5704) and sotrovimab (n = 3322) between February and November 2022, when Omicron subvariants BA.2, BA.5, or BQ.1 were dominant [41].

A recent metanalysis of 14 studies including 41,000 patients who received sotrovimab (in US, UK, Italy, Denmark, France, Qatar, and Japan), which included four studies comparing the effectiveness of sotrovimab with untreated or no monoclonal antibody treatment controls, two studies comparing sotrovimab with other treatments, three single-arm studies comparing outcomes during BA.2 and/or BA.5 versus BA.1, and five studies reporting rates of clinical outcomes in patients treated with sotrovimab, it was reported that the rates of COVID-19-related hospitalization or mortality among sotrovimab-treated patients were consistently low (0.95% to 4.0% during BA.2; 0.5% to 2.0% during BA.5). All-cause hospitalization or mortality was also low in these patients (1.7% to 2.0% during BA.2; 3.4% during combined BA.2 and BA.5 periods). During BA.2, a lower risk of all-cause hospitalization or mortality was reported across studies with sotrovimab versus untreated cohorts. Compared with other treatments, sotrovimab was associated with a lower (molnupiravir) or similar (nirmatrelvir/ritonavir) risk of COVID-19-related hospitalization or mortality during BA.2 and BA.5, and there was no significant difference in outcomes between the BA.1, BA.2, and BA.5 periods [42].

## 4. Discussion

Sotrovimab proved to be the most resistance-resilient anti-Spike mAb monotherapy during the course of the COVID-19 pandemic, largely because it targeted a very conserved epitope which rarely mutates. Despite sotrovimab use being first limited by the United States FDA on 30 March 2022, and then deauthorized on 5 April 2022 due to inefficacy against BA.2, its usage largely continued in both the US [43] and the EU [44]. Figure 1 shows how usage continued in England. In the absence of other effective anti-Spike mAb therapies, some clinicians advocated the continued use of sotrovimab against omicron lineages, even though the IC_50_ against these variants was never below 500 ng/mL. It should be nevertheless noted that the widespread vaccine boosting campaign, which results in antibody responses in most individuals, has minimized the additional benefits conveyed by early treatment, bringing into question the cost-effectiveness of the approach.

Observational and real-world data reports of continued sotrovimab efficacy, despite a precipitous loss of binding to later variants, are difficult to reconcile with the established principles of antibody action, which require binding to the virion for neutralization and activation of Fc-mediated antiviral functions. Assuming that those beneficial effects are real, unlikely but possible explanations include the persistence of minoritarian sotrovimab-susceptible populations in some individuals, insufficient sampling of VOC prevalence, or some as-yet uncharacterized effect of the mAb on the immune system that affected immune function. Recently, a defect in post-infection B-cell memory generation after treatment with bamlanivimab has been reported for the epitopes targeted by this mAb [45]. Whether the same concerns apply to other anti-Spike mAbs, such as sotrovimab, remains to be investigated and could represent a clinical concern. With the current BA.2.86* wave originating in November 2023, sotrovimab has now totally lost its in vitro efficacy. While its efficacy could conceivably return in the future with a novel viral lineage that again uses the sequences that defined its epitope, it seems prudent to invest in the pipeline and to work on designing combinations of mAbs that are less susceptible to the emergence of mutations [35]. In this regard, VIR-7832 is a modification of sotrovimab with the addition of a three-amino acid mutation GAALIE (G236A, A330L, I332E) to the Fc region which enhances binding to FcγRIIa and FcγRIIIa, decreases affinity for FcγRIIb in vitro, and evoke protective CD8^+^ T lymphocytes in vivo [46,47]. However, VIR-7832 never reached clinical use.

In summary, sotrovimab was a success story that nonetheless provides a cautionary tale of how even a superbly designed mAb remains vulnerable to rapid viral evolution. In fact, the concept of using long half-life mAbs as treatment for immunocompromised patients who are unable to mount their own antibody responses, while rational and successful for some time, may carry within it the seeds of eventual failure. These patients harbor swarms of variants, and the introduction of monotherapy with an mAb will invariably select for variants that do not demonstrate antibody-mediated antiviral effects [31]. This phenomenon was carefully documented in a patient who received sotrovimab, which led to the emergence of mAb-resistant variants [48].

Sotrovimab illustrates the promise of antiviral mAbs for providing long-term passive immunity while also highlighting the limitations of this approach. Going forward, we need to learn how to use these promising therapies more effectively by reducing the likelihood of resistance as is carried out in some types of antimicrobial chemotherapy, where drug combinations are used to reduce the likelihood of the emergence of resistance. In this regard, using combinations of mAbs, possibly with concomitant small molecule antiviral therapy, could prolong the useful lives of these remarkable immunoglobulin reagents. Sotrovimab was a product of the great advances in molecular biology, immunology, and virology during the past half century but proved vulnerable to viral evolution; this vulnerability was exacerbated by the manner in which it was used clinically. Going forward, we need comparable advances in clinical practice that consider the biology of the system to optimize the future use of immunoglobulin therapeutics.

**Table 1:** In vitro efficacy of sotrovimab at neutralizing selected SARS-CoV-2 VOCs. Median fold-reduction in neuralization titers compared to wild-type was retrieved from the Stanford Antiviral Resistance database (accessed at https://covdb.stanford.edu/search-drdb/?antibodies=Sotrovimab, on 24 January 2024). Please note that the 50% inhibitory concentration (IC_50_) does not account for heterogeneity of viral inoculum (live authentic vs. pseudoviruses), viral dose, replication-competent cells, and detection methods.

WHO VOC	PANGOLIN Name	NextStrain Name	UKHSA/PHE Name	Nick Name	Median Fold-Reduction in Neutralization Titers Compared to Wild-Type	IC_50_ (ng/mL) from Selected Studies
wild-type	B.1	-	-	-	-	140 [49], 200 [50], 100 [51], 1000 [52], 58 [53], 94 [54], 27 [55], 32 [56]
Alpha	B.1.1.7	20I/S:501Y.V1	VOC-20DEC-01	-	1.8	187.2 [51], 50 [57], 80 [53], 81 [56]
Beta	B.1.351	20H/S:501Y.V2	VOC-20DEC-02	-	1	71.9 [51], 50 [58], 50 [53], 31 [56]
Gamma	P.1	20J/S:501Y.V3	VOC-21JAN-02	-	1	73.11 [51], 66 [53]
Delta	B.1.617.2	21A/S:478K and descendants 21I/21J	VUI-21APR02	-	1.1	51.3 [51], 73 [53], 42 [56]
Kappa	B.1.617.1	21B	-	-	-	119 [51]
Omicron	BA.1	21K (descendant of 21M)	VUI-21NOV-01	-	3.8	340 [49], 169.2 [51], 181 [53], 138 [56]
	BA.1.1	-	-	-	2.7	165 [51], 130 [56]
	BA.2	21L (descendant of 21M)	VUI-22JAN-01	-	20	1507 [50], 2090 [49], 972.8 [51], 559 [59], 559 [59], 2190 [54], 1240 [56]
	BA.2.12.1	22C	-	-	20	629 [54], 1035 [56]
	BA.2.75	22D	-	Centaurus	12	436 [55], 960 [56]
	BA.4 BA.5	22A 22B	VOC-22APR-03 VOC-22APR-04	-	22	1260 [49], 1261 [54], 577 [55], 1120 [56]
	BF.7	-	-	Minotaur	48	1520 [56]
	CH.1.1	-	-	Orthrus	16	437 [55], 711 [56]
	BQ.1.1	22E	V-22OCT-01	Cerberus	118	4263 [56]
	XBB.1.5	23A	V-23JAN-01	Kraken	15	970 [11], 416 [59], 338 [59], 900 [60], 1300 [49], 970 [11,61], 575 [56]
	XBB.1.5 + E554K	-	-	-	-	950 [60]
	XBB.1.5 + L455F	-	-	-	-	740 [49], 880 [61]
	XBB.1.5 + F456L	-	-	-	-	1170 [49], 880 [61]
	XBB.1.5 + FLip	-	-	-	-	5500 [49], 1020 [61]
	XBB.1.9.1	23D	-	Hyperion	-	>50,000 [62]
	XBB.1.16	23B	V-23APR-01	Arcturus	9.7	780 [63], >3840 [64]
	XBB.1.16.1	-	-	-	-	>10,000 [52]
	EG.5.1	23F	V-23JUL-01	Eris	2.7	532 [50], 880 [11] (EG.5), 1130 [49]
	EG.5.1.3	-	-	-	5	5000 [52]
	BA.2.86	23I	V-23AUG-01	Pirola clan	>5	26,042 [50], 1890 [11,60], >10,000 [59], >12,000 [49]
	BA.2.86.1	-	-		>10	>10,000 [52]
	JN.1	-	-		-	2300 [11]
	JD.1.1	-	-	FLip’s	-	1030 [11]
	HV.1	-	-		-	1190 [11]
	HK.3	23H	-		-	1220 [11]

**Table 2:** Spike mutations associated with sotrovimab resistance in in vitro studies. Bold characters show the ones that have been detected as emerging in vivo after treatment with the specific mAb. In cases where the exact amino acid change has not been studied in vitro, only the residue is highlighted. Number within parentheses represent the median fold-reduction in neutralizing antibody titers.

Spike Mutation	Reference
S:**337**H (6)	[51,65]
S:**337**L (180)	[51,65]
S:**337**R (>192)	[51,65]
S:**337**T (7)	[51,65]
S:**340**A (>100)	[51,65]
S:**340**D (12)	[51,66]
S:**340**G (22)	[51,65]
S:**340**K (>297)	[51,65,66,67]
S:**340**Q (>50)	[51]
S:**340**V (>200)	[51]
S:**356**T (5.9)	[51]
S:**371**F (13)	[68,69,70]
S:**371**L (12)	[70]
S:377K (>704)	[51]

**Table 3:** Reported cases of sotrovimab treatment-emergent resistance.

Reference	Incidence of Treatment-Emergent Resistance	SARS-CoV-2 Sublineage	Treatment-Emergent Spike Mutations
Rockett et al. [24]	4 cases out of 100 (4%)	Delta	E340K/A/V
Birnie et al. [25]	10 cases out of 18 (55.6%) (15 immunocompromised patients)	BA.1 (94%) BA.2 (6%)	E340K/A/V/D/G/Q, P337L/R/S
Focosi et al. [26]	3 cases out of 16 (18.8%) immunocompromised patients	2 BA.1, 1 BA.2	E340D
Vellas et al. [27]	18 cases out of 34 (52.9%) immunocompromised patients	17 BA.1 1 BA.2	P337L/S, E340A/K/D/G, K356T, S371F
Huygens et al. [28]	4 cases of 25 (16%) BA.1-infected patients; 2 cases of 7 (28.6%) BA.2-infected	BA.1 BA.2	P337X, E340X
Andrés et al. [29]	5 cases out of 8 (62.5%) immunocompromised patients	BA.1 (7) AY.100 (1)	P337L, E340D/R/K/V/Q, R346T, K356T
Destras et al. [30]	8 patients	BA.1	P337R/S, E340A/D/K/Q
Gupta et al. [31]	9 cases out of 34 (26.5%)	BA.1.1 (n = 14) BA.1 (n = 13) BA.2 (n = 7)	E340K/D/V
Ragonnet-Cronin et al. [32]	54 out of 134 (40.3%) patients	Delta, BA.1, BA.2	P337R/S, E340A/D/K/V, K356T
Palomino-Cabrera et al. [33]	15 out of 22 (68%) patients	BA.5	P337S/R/T/L/A/H, E340Q/A/D/K/V/G, R346T and K356T
Mazzetti et al. [34]	1 immunocompromised patient	BA.1.1.16	E340A
Gliga et al. [66]	14 out of 43 (32.6%) patients	BA.1	P337S/H/L/R, E340D/K/V
Leducq et al. [71]	47 out of 166 patients (131 immunocompromised)	BA.1 (61%) BA.2 (39%)	P337S/R/L/H (10%) K356T/R (13%) E340D/K/A/Q/V/G (10%)
Hirotsu et al. [48]	1 immunocompromised patient	BA.1.1	P337L and E340K
Subramanian et al. [12]	35 out of 170 (20.6%) patients		P337L, E340A/K/V, and C361T
